# PD-1/PD-L1 pathway relieves incision induced acute postoperative pain via inhibiting the neuroinflammation in dorsal root ganglion of rats

**DOI:** 10.1038/s41598-025-31618-6

**Published:** 2025-12-17

**Authors:** Honglei Zhang, Yi Wang, Shiwei Wen, Jing Li

**Affiliations:** 1 Department of Anesthesiology, Affiliated Hospital of Zunyi Medical University, 149 Dalian Street, Zunyi, 563000 Guizhou China; 2 Department of Operating Room, Affiliated Hospital of Zunyi Medical University, 149 Dalian Street, Zunyi, 563000 Guizhou China

**Keywords:** Acute postoperative pain, PD-1, PD-L1, Neuroinflammation, Treatment, Cell biology, Immunology, Molecular biology

## Abstract

Acute postoperative pain represents a significant clinical challenge, yet its underlying mechanisms remain incompletely understood. A previous study found that PD-1/PD-L1can relieve chronic pain. Mechanical withdrawal thresholds (MWT) and thermal withdrawal latency (TWL) were quantified in rats at baseline (pre-incision) and at postoperative timepoints of 2, 8, and 24 h. Concurrently, PD-1/PD-L1 expression levels and neuroinflammatory markers were assessed at the 24-hour terminal timepoint. PD-1 inhibitors and agonists were administered via intrathecal injection immediately after surgical incision and at 12 h postoperatively, respectively. It was found that incision surgery resulted in significant postoperative pain accompanied by upregulation of PD-1/PD-L1 and neuroinflammation. Inhibition of PD-1 expression could further exacerbate acute postoperative pain by upregulating neuroinflammation, whereas upregulation of PD-1 expression could alleviate acute postoperative pain by inhibiting neuroinflammation. Activation of the PD-1/PD-L1 pathway relieves acute postoperative pain induced by plantar incision. This may be a therapeutic target for acute postoperative pain.

**Supplementary Information:**

The online version contains supplementary material available at 10.1038/s41598-025-31618-6.

## Introduction

With the increase in surgical volume, postoperative pain has become a globally important issue that seriously affects patients’ postoperative recovery. Acute postoperative pain, if not managed properly, carries the risk of progressing into chronic pain^1,2^. In addition to pain, acute pain can also lead to postoperative delirium and cognitive dysfunction in patients^3^. Therefore, effective management acute postoperative pain is particularly important. Currently, opioids and antipyretic anti-inflammatory drugs are still mainly used for analgesia worldwide. Both of them have significant side effects, creating an urgent need to develop new postoperative analgesic drugs with fewer side effects.

The programmed cell death-1 (PD-1) is a type I membrane protein with 288 amino acids, a member of the expanded CD28/CTLA-4 family of T cell modulators^4^. The Programmed cell death ligand 1 (PD-L1 or *Pdcd1lg1*, CD274, B7-H1) is a 290-amino acid protein^5^. PD-1 and its ligand PD-L1, as important immunomodulatory factors, play an important role in tumor therapy^6,7^. Recently, several studies found that PD-1/PD-L1 also can regulate pain through immunity and neuroinflammation^8,9^. Chen et al. reported that PD-L1 can activate PD-1 to inhibit inflammatory pain and chronic neuropathic pain after nerve injury^10^. PD-1/PD-L1 inhibition enhances chemotherapy-induced neuropathic pain by suppressing neuroimmune antinociceptive signaling^8^. However, whether PD-1/PD-L1 can alleviate acute postoperative pain has not been studied. Additionally, the dorsal root ganglion (DRG) is a primary neuron for sensory transmission and has become an important target for pain treatment.

In this study, we hypothesized that PD-1/PD-L1 pathway is involved in the regulation of acute postoperative pain induced by plantar incision. The results of this experiment demonstrated that the activation of PD-1/PD-L1 signaling pathway could reduce acute pain and inhibit neuroinflammatory response after plantar incision in rats.

## Materials and methods

### Animals

This study was approved by the Animal Management and Use Committee of Zunyi Medical University (ZMU21-2503-039). Animal studies were conducted in accordance with the Guidelines for the Management and Use of Laboratory Animals in China. Male Sprague-Dawley rats (6–8 weeks old, 200–250 g) were purchased from Changsha Tianqin Biotechnology (Hunan, China). Three - four rats were housed in cages at a constant room temperature of 23 ± 2 °C and a relative humidity of 55% ± 2% on a 12 h light/dark cycle. Food and water were freely available. All mice were euthanized by inhalation excessive amounts of the volatile anesthetic gas isoflurane, resulting in deep anesthesia until respiratory and cardiac arrest.

### Plantar incision pain model

Plantar incision pain model was established as previously described^11^. Rats were anesthetized with 2.0–3.0% isoflurane (Reward, Shenzhen, China). Then, after being sterilized with iodophor, a 1-cm incision was made in the plantar aspect of the right hind paw 0.5 cm from the end of the heel. The skin, fascia and flexor muscles below were incised sequentially. Blunt curved forceps were inserted into the short flexor muscles of the toes, and the muscles were separated and retracted. After adequate hemostasis, the wound was closed with a 5 − 0 nylon suture. Subsequently, topical antibiotics were given to prevent wound infection. Control rats were anesthetized under the same conditions without incision.

### Experiment design and intrathecal injection

Rats were randomly divided into four groups: control group, incision group, incision + solvent, and incision + BMS-1 (incision + H-20). BMS-1 and H-20 were administered once immediately after modeling and 12 h after surgery. All six groups of rats were anesthetized with 2–3% isoflurane. BMS-1 (MCE, Monmouth Junction, NJ, USA) and H-20 (MCE, Monmouth Junction, NJ, USA) were administered by intrathecal injection. The dissolution method and intrathecal injection method of BMS-1 and H-20 are described in the instructions on the official website of MCE. The reagents (20–50 µl) were injected into the cerebrospinal fluid between the L5 and L 6 intervertebral spaces via intrathecal injection using a microsyringe (High Pigeon, Shanghai, China)^12^. Once the puncture needle was successfully inserted into the subarachnoid space, a sudden jerk of the tail is often observed.

### Pain behavioral tests

Mechanical withdrawal threshold (MWT) and thermal withdrawal latency (TWL) of the plantar of the ipsilateral side were measured before incision surgery and at 2, 8, and 24 h after incision surgery.

### MWT

MWT were measured as previously described^12^. First the rats were placed in the experimental environment for half an hour to acclimatize. Von Frey filaments are used to measure the MWT. The filaments were used to apply a vertical force from small to large scale (0.16–26 g) to the right paw of the rat. Each filament was applied for 4–6 s. A positive response was defined as lifting, flinching, or licking of the paw upon stimulation in at least 3 out of 5 applications. The up-down method was employed: If a positive reaction was observed, a filament with the next lower force is applied. If no reaction occurs, a filament with the next higher force is used. The minimum force required for a positive reaction was recorded as the MWT.

### TWL

As mentioned above, the TWL was measured^12^. Rats were housed separately in an glass enclosure on a transparent glass surface at 25 °C. After 30 min of adaptation, the plantar radiant heat pain tester (Ugo Basile, Italy) was used for measurement. The right plantar of rats was directly irradiated by constant heat method. When the rats showed foot contraction, foot lifting and foot licking, it was considered as a positive reaction. Each rat was performed 5 times with an interval of 5 min. The average value of the five tests was considered to the TWL.

### Western blot (WB)

The ipsilateral L4-L6 DRG were quickly harvested 24 h after incision surgery and stored at -80 °C. Tissues were homogenized in RIPA buffer (Beyotime, Beijing, China) containing protease inhibitors (Roche, Shanghai, China). The homogenates were kept on ice for 20 min and centrifuged at 12,000 rpm for 20 min. The tissue supernatant was taken. Protein quantification was performed using the BCA (Beyotime, Beijing, China) method and the protein concentration was adjusted. The protein is then electrophoresed and transferred to a PVDF membrane (Millipore, Massachusetts, USA). After washed with tris-borate-sodium tween-20 (TBST) for 3 times (10 min each time), the strips were added the primary antibody: anti-β-actin (1:1000; Abcam, Cambridge, UK), anti-PD-1 (1:2000, Proteintech, Wuhan, China) and anti-PD-L1 (1:2000, Proteintech, Wuhan, China). Then these trips were placed in a refrigerator at 4 ℃ overnight. After washed 3 times by TBST, the strips were incubated with secondary antibodies for 2 h. Finally, the strips were exposed.

### Elisa

General anesthesia was administered to rats by intraperitoneal injection of sodium pentobarbital (50 mg/kg), and ipsilateral L4-L6 DRG tissues were quickly taken and frozen at -80 °C until use. The tissues were rinsed with pre-cooled PBS, and then were sheared. The sheared tissue was added to a glass homogenizer with an appropriate amount of PBS, and ultrasonicated thoroughly. Finally, the homogenate was centrifuged at 4 °C, 5000 × g for 10 min, and the supernatant was taken for testing. Briefly, the standard solution was prepared as instructed, and the tissue supernatants were diluted appropriately. Then, 100 µL of standard and sample dilution were added sequentially according to the instructions, 100 µL of biotinylated antibody working solution was added to each well, and the enzyme plate with laminating film was incubated at 37℃ for 1 h. After incubation, each well was added 350 µL of washing solution, and then added 100 µL of HRP enzyme conjugate working solution. The enzyme plate was covered with membrane, and incubated at 37℃ for 30 min. After shaking and washing for 5 times, 90 µL of substrate solution (TMB) was added to each well, and the plate was covered with a membrane and incubated at 37℃ for about 15 min without light. Finally, 50 µL of termination solution was added to terminate the reaction, and the optical density (OD) of each well was measured at 450 nm using an enzyme counter.

### Immunofluorescence

Twenty-four hours after incision surgery, rats were transcardially perfused, and the DRG was dissected and fixed in 4% paraformaldehyde (PFA) for 24 h. Then the tissues were dehydrated in 30% high glucose. The embedding racks with tissues were placed into the hanging basket of a sterilizer (Zhongwei, Changzhou, China) and dehydrated sequentially with gradient ethanol in the sterilizer. The wax-impregnated tissues were embedded in the embedder (Changzhou Zhongwei, China), and the wax blocks were sliced in a paraffin slicer (ThermoFisher, Massachusetts, USA) at a thickness of 4 μm. Sections were now baked in a 37℃ oven for 2 h and then dewaxed in xylene and gradient ethanol. The dewaxed slices were sealed in 5% BSA and 0.3% triton X-100 for 2 h, and then applied with primary antibody overnight at 4℃ refrigerators: anti-PD-1 (1:500, Proteintech, Wuhan, China), anti-CGRP (1:200; Santa, California, USA), anti-IB4 (1:200; Sigma, Louis Missouri, USA) and anti-NF-200 (1:200; Cell Signaling Technology, Boston, USA). The next day, the slides were washed 3 times with PBS, and then incubated with secondary antibody for 2 h: goat anti-rabbit IgG H & L (1: 1000; Abcam, Cambridge, UK), goat anti-mouse IgG H & L (1: 1000; Abcam, Cambridge, UK). After washed again with PBS, the slices were sealed with an anti-quenching sealer (SouthernBiotech, Alabama, USA).

### Statistical analysis

All data were statistically analyzed using GraphPad Prism 11.0 (GraphPad Software, La Jolla, CA). Data were expressed as mean ± SD. The statistical difference between the two groups was analyzed by unpaired t test. The statistical differences between the two groups at multiple time points were analyzed by two-way analysis of variance, and then the multiple comparison test was performed. *P* < 0.05 was considered statistically significant. Immunofluorescence images were analyzed using Image J v.1.8 (NIH, Washington, USA).

## Results

### Incision pain leads to up-regulation of the PD-1/PD-L1 pathway, accompanied with upregulation of neuroinflammation

**Fig. 1:**
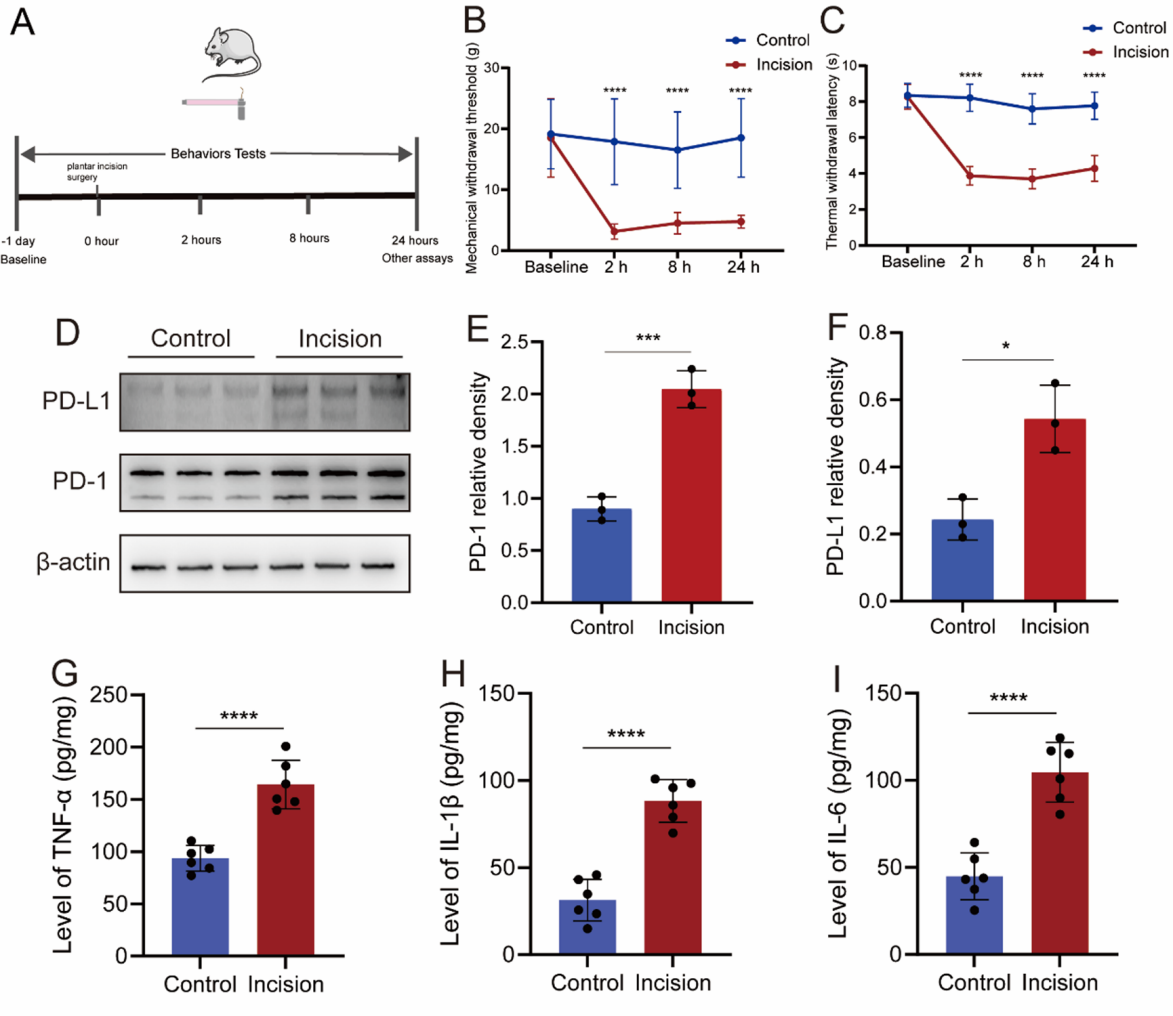
Incision pain leads to up-regulation of the PD-1/PD-L1 pathway, accompanied with upregulation of neuroinflammation. (**A**) Experimental flow chart. (**B**) MWT decreased 2 h, 8 h and 24 h after incision surgery. *n* = 12. (**C**) TWL decreased 2 h, 8 h and 24 h after incision surgery. *n* = 8. (**D**) The WB test results of PD-1 and PD-L1. *n* = 3. (**E**) The statistical result of PD-1expression. (**F**) The statistical result of PD-L1expression. (**G**) The expression of TNF-α. (**H**) The expression of IL-1β. *n* = 6. (**I**) The expression of IL-6. *P < 0.05, ***P < 0.001, ****P < 0.0001. Two-way of ANOVA tests and Sidak’s multiple comparisons test was used for statistical behavioral results and unpaired t tests were used for the WB and Elisa.

MWT and TWL were assessed before incision surgery, 2 h, 8 h and 24 h after incision surgery. MWT and TWL significantly decreased 2 h after incision surgery and remained low at 24 h postoperatively (Fig. 1A-C). It has found that both PD-1 and PD-L1 up-regulated in DRG (Fig. 1D-F). Concomitantly, the expression of TNF-α, IL-1β and IL-6 up-regulated (Fig. 1G-I).

**Fig. 2:**
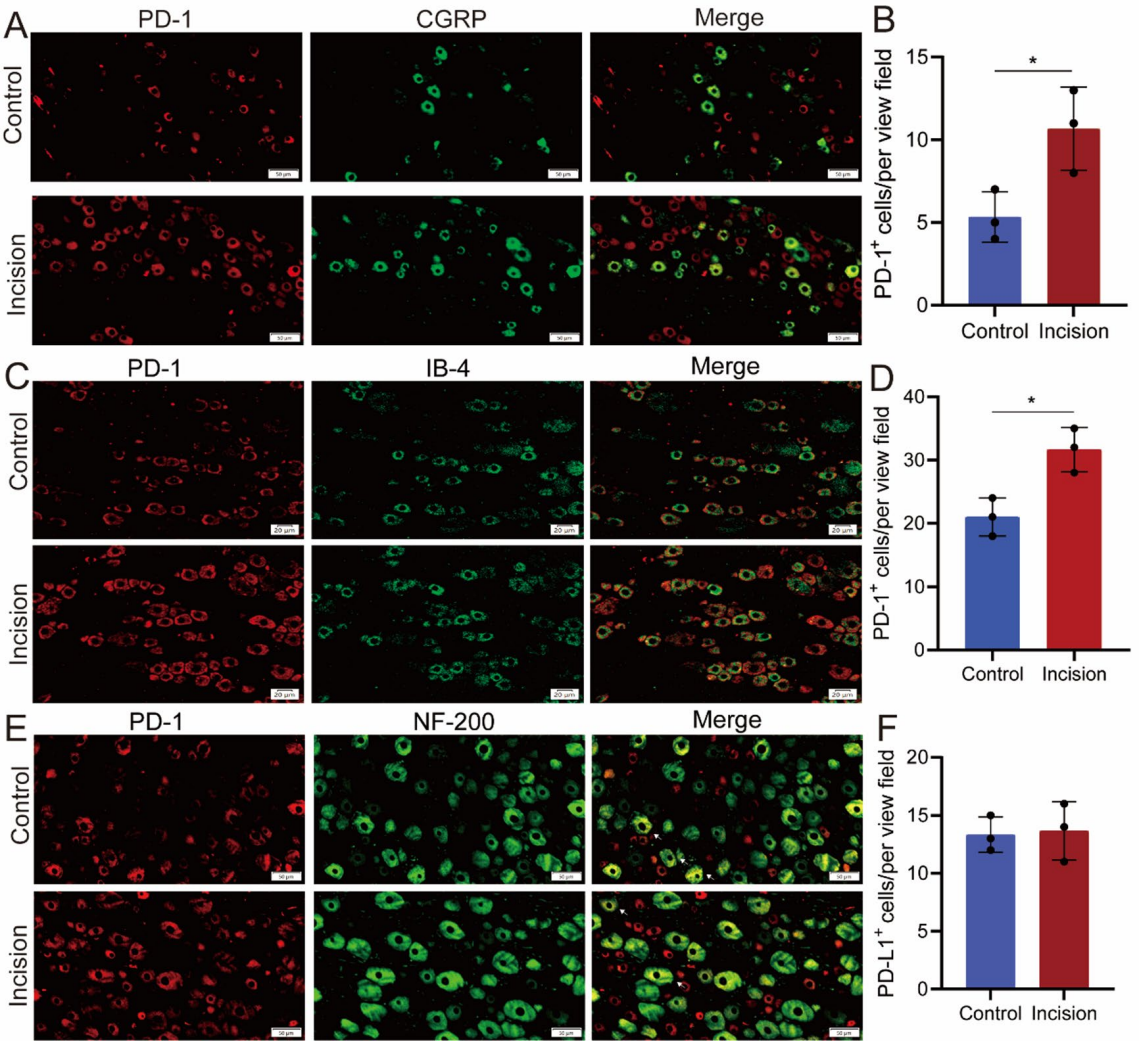
PD-1 expression in DRG neurons. (**A**) Representative image of immunofluorescence co-staining of PD-1 with CGRP. Scale bar: 50 μm. (**B**) Number of the immunofluorescence cells of co-location of PD-1 with CGRP in A. *n* = 3. (**C**) Representative image of immunofluorescence co-staining of PD-1 with IB4. Scale bar: 20 μm. (**D**) Number of the immunofluorescence cells of co-location of PD-1 with IB4 in C. *n* = 3. (**E**) Representative image of immunofluorescence co-staining of PD-1 with NF-200. Scale bar: 50 μm. (**F**) Number of the immunofluorescence cells of co-location of PD-1 with NF-200 in E. *n* = 3. *P < 0.05. Unpaired t tests were used for the immunofluorescence.

In addition, immunofluorescence results revealed that upregulated PD-1 was mainly expressed on CGRP^+^ and IB4^+^ neurons in DRG (Fig. 2A-D). While, there was no significant increase in NF-200 neurons in DRG (Fig. 2E-F).

### **Inhibition of PD-1 expression exacerbates incision pain by upregulating neuroinflammation**

**Fig. 3:**
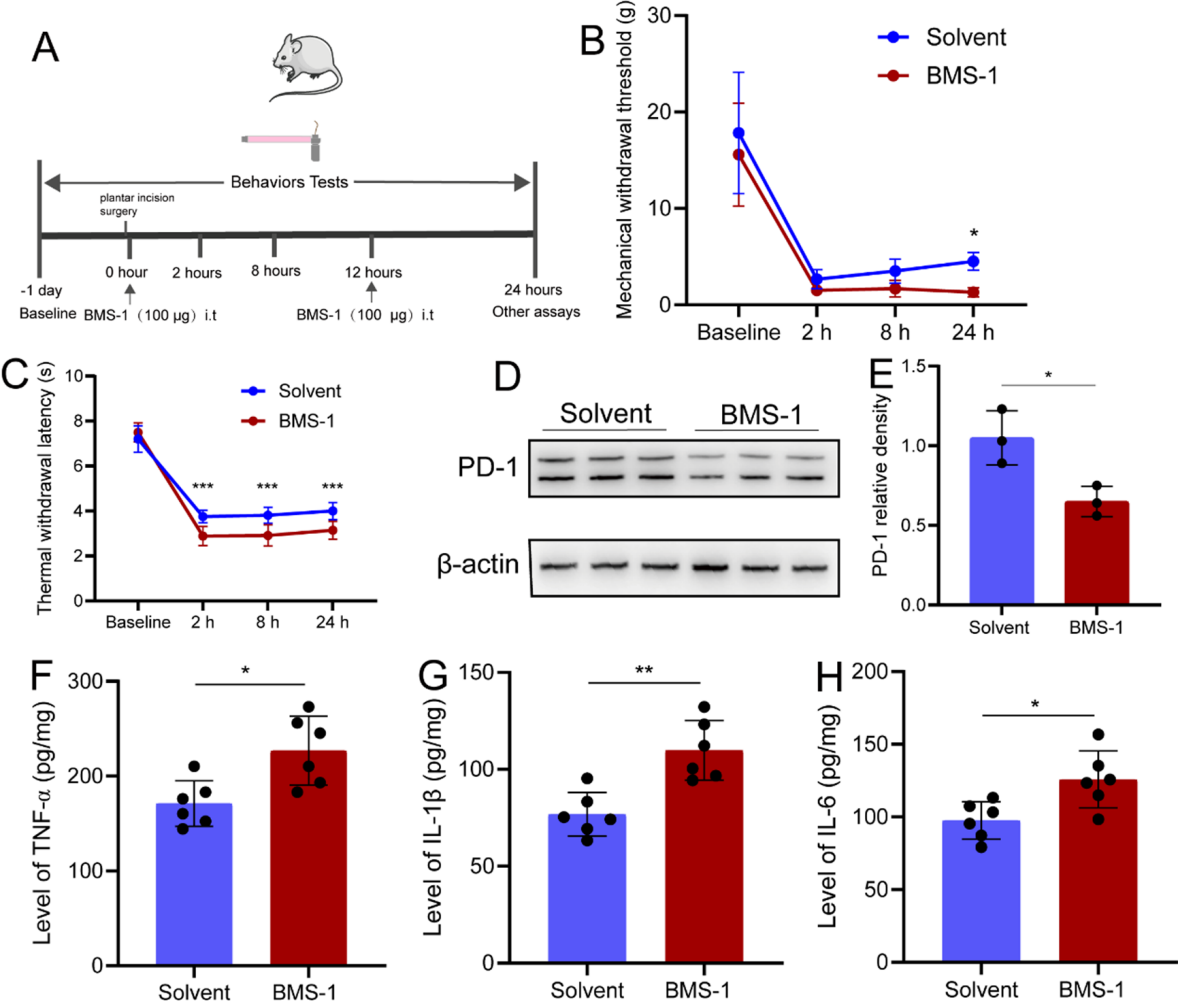
BMS-1 exacerbates incision pain by up-regulating neuroinflammation. (**A**) Experimental flow chart. (**B**) BMS-1 induced further lower MWT. *n* = 12. (**C**) BMS-1 induced further lower TWL. *n* = 8. (**D**) The WB test results of PD-1. (**E**) The statistical result of PD-1expression. *n* = 3. (**F**) The statistical result of TNF-α expression. *n* = 6. (**G**) The statistical result of IL-1β expression. *n* = 6. (**H**) The statistical result of IL-6 expression. *n* = 6. *P < 0.05, **P < 0.01, ***P < 0.001. Two-way of ANOVA tests and Sidak’s multiple comparisons test was used for statistical behavioral results and unpaired t tests were used for the WB and Elisa.

BMS-1 was used to inhibit the expression of PD-1^13^. It was found that BMS-1 (100 µg i.t) could further exacerbates incision induced acute postoperative pain. Compared with the solvent group, the MWT and TWL in the BMS-1 group further decreased (Fig. 3A-C). Besides, the expression of PD-1 was significantly inhibited in DRG (Fig. 3D-E). It was also found that after inhibition the expression of PD-1, the expression of TNF-α, IL-1β and IL-6 also was further up-regulated (Fig. 3F-H).

**Fig. 4:**
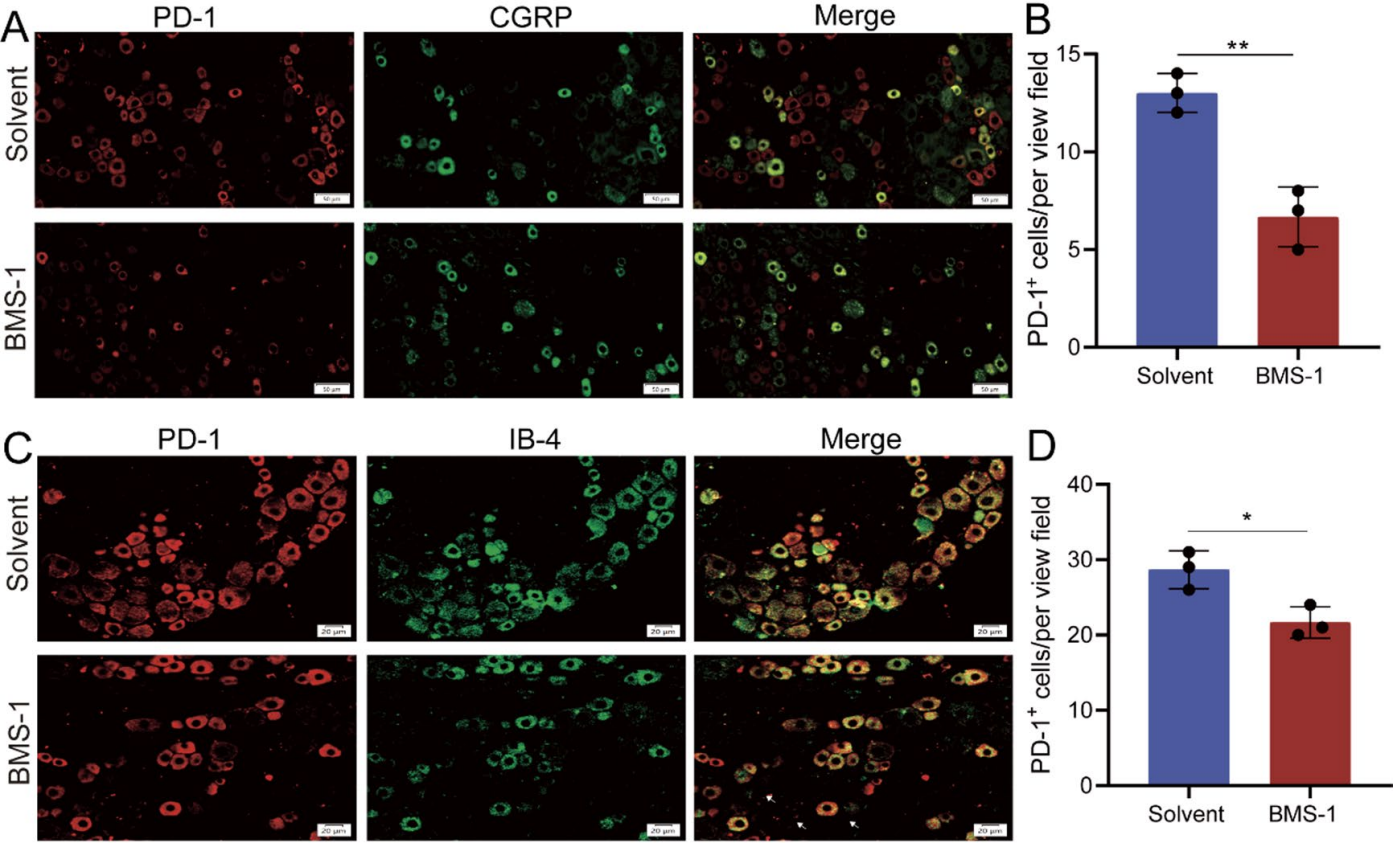
BMS-1 inhibits the neuronal type of PD-1 expression. (**A**) Representative image of immunofluorescence co-staining of PD-1 with CGRP. Scale bar: 50 μm. (**B**) Number of the immunofluorescence cells of co-location of PD-1 with CGRP in A. *n* = 3. (**C**) Representative image of immunofluorescence co-staining of PD-1 with IB4. Scale bar: 20 μm. (**D**) Number of the immunofluorescence cells of co-location of PD-1 with IB4 in C. *n* = 3. *P < 0.05, **P < 0.01. Unpaired t tests were used for the immunofluorescence.

We further explored the inhibitory effect of BMS-1 on PD-1 in DRG neurons. Immunohistochemical analysis revealed a significant inhibition of PD-1 expression in both CGRP^+^ and IB4^+^ neurons (Fig. 4A-D).

### Agonistic PD-1 relieves incision pain by down-regulating neuroinflammation

**Fig. 5:**
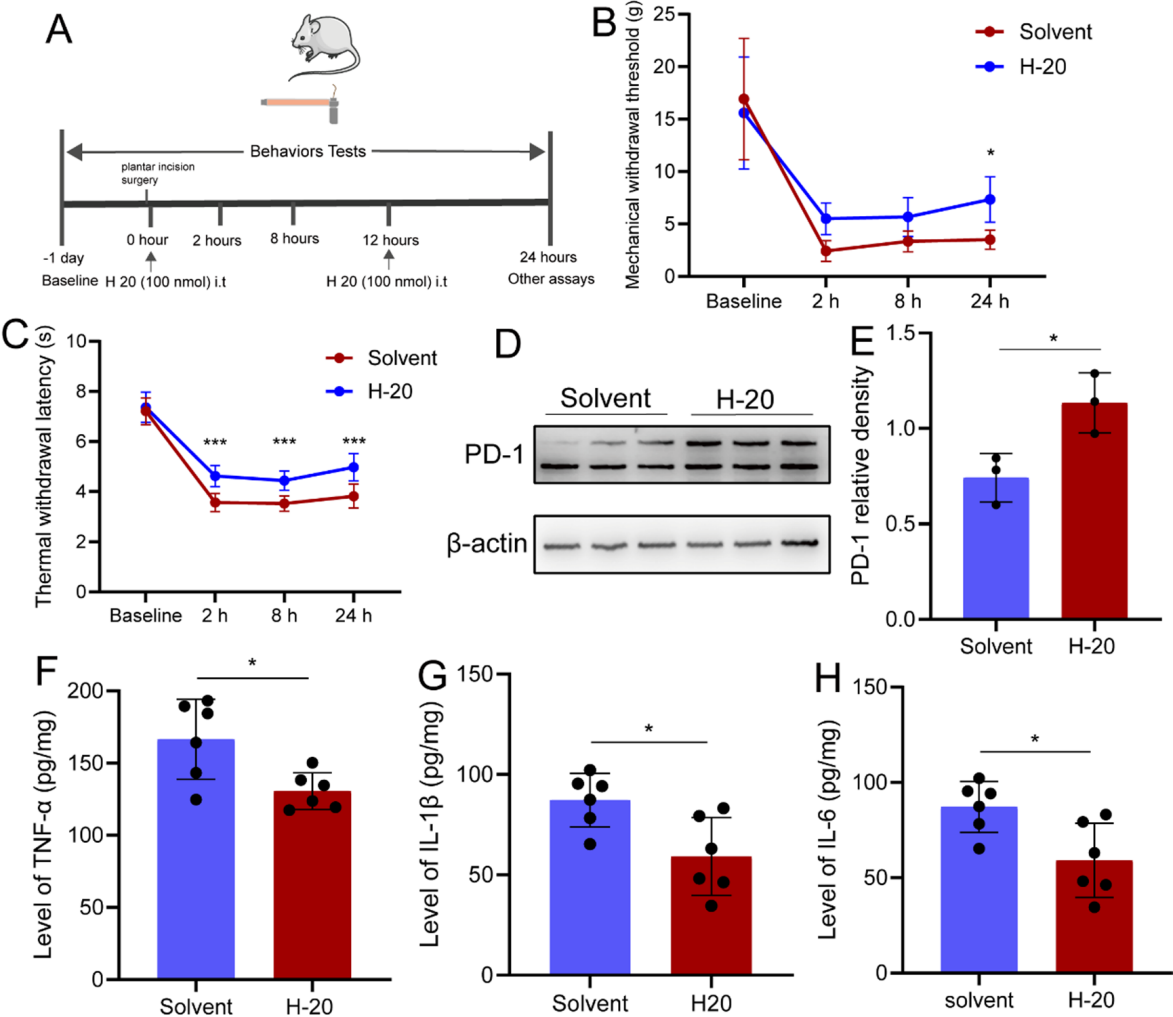
H-20 relieve incision pain by down-regulating neuroinflammation. (**A**) Experimental flow chart. (**B**) H-20 induced higher MWT. *n* = 12. (**C**) H-20 induced higher TWL. *n* = 8. (**D**) The WB test results of PD-1. (**E**) The statistical result of PD-1expression. *n* = 3. (**F**) The statistical result of TNF-α expression. *n* = 6. (**G**) The statistical result of IL-1β expression. *n* = 6. (**H**) The statistical result of IL-6 expression. *n* = 6. *P < 0.05, ***P < 0.001. Two-way of ANOVA tests and Sidak’s multiple comparisons test was used for statistical behavioral results and unpaired t tests were used for the WB and Elisa.

It has been reported that H-20 can relieve chronic pian^14^. In this study, it was found that H-20 (100 nmol i.t) also can relieve acute postoperative pain. Compared with the solvent group, both of the MWT and TWL in the H-20 group increased (Fig. 5A-C). In addition, the expression of PD-1 also up-regulated (Fig. 5D-E). While the expression of TNF-α, IL-1β and IL-6 was down-regulated (Fig. 5F-H).

**Fig. 6:**
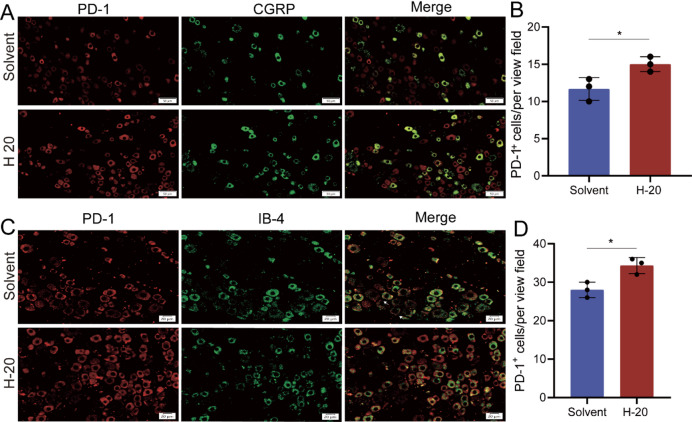
H-20 significantly upregulated PD-1 expression specifically in CGRP^+^ and IB4^+^ neuron in DRG. (**A**) Representative image of immunofluorescence co-staining of PD-1 with CGRP. Scale bar: 50 μm. (**B**) Number of the immunofluorescence cells of co-location of PD-1 with CGRP in A. *n* = 3. (**C**) Representative image of immunofluorescence co-staining of PD-1 with IB4. Scale bar: 20 μm. (**D**) Number of the immunofluorescence cells of co-location of PD-1 with IB4 in C. *n* = 3. *P < 0.05. Unpaired t tests were used for the immunofluorescence.

We further investigated the specific subtypes of DRG neurons in which H-20 induces PD-1 upregulation. It was found that the up-regulation of PD-1 was expressed in CGRP^+^ and IB4^+^ neuron (Fig. 6A-D).

## Discussion

In this study, w we demonstrated that the incision pain model led to the up-regulation of PD-1/PD-L1 in DRG concomitant with neuroinflammation. Inhibition of PD-1/PD-L1 expression in DRG can further aggravate postoperative acute pain via enhancing neuroinflammation; however, activation of PD-1/PD-L1 expression can alleviate incision pain by inhibiting neuroinflammation.

It has been reported that PD-1/PD-L1 is involved in pain regulation. Compared with wild-type mice, PD-L1 knockout mice had increased baseline pain sensitivity to thermal and mechanical stimulation^10^. Intraplantar or intrathecal injection of PD-L1 was sufficient to increase the pain threshold in non-injured naïve mice^10^. It has reported that PD-1 inhibits chronic pain by suppressing nociceptive neuron activity via PD-1^10^. PD-1/PD-L1 inhibition enhances chemotherapy-induced neuropathic pain by suppressing neuroimmune antinociceptive signaling, indicating that PD-1/PD-L1 can relieve chemotherapy-induced neuropathic pain^8^. PD-1-deficient mice exhibit enhanced inflammation and neuropathic pain via immune modulation following nerve injury^15^. PD-L1 and PD-1 expressed in trigeminal ganglia may inhibit pain in an acute migraine model^16^. PD-1 Regulates GABAergic Neurotransmission and GABA-Mediated Analgesia and Anesthesia^17^. Deng et al. found that electroacupuncture alleviates the complete Freund’s adjuvant (CFA) induced inflammatory pain via PD-L1/PD-1-SHP-1 pathway^13^. Consistent with these findings, we found that incision surgery can up-regulate the expression of PD-1 and PD-L1. Inhibition of PD-1/PD-L1 exacerbates incision induced postoperative pain, whereas activation PD-1/PD-L1 can relieve the pain, indicating that incision pain induces increased PD-1/PD-L1 expression, which may be a compensatory mechanism. Agitation of PD-1/PD-L1 further relieves incision pain. Therefore, PD-1/PD-L1 maybe the vital target for acute postoperative pain.

PD-1/PD-L1 is an important regulator in immunity^18–20^. Previous research has established the PD-1/PD-L1 pathway as a critical regulator of neuroinflammation within the central nervous system (CNS), where it operates primarily in glial cells, such as microglia and astrocytes, to suppress immune activation^21–25^. In contrast, our study unveils a distinct site and mechanism of action in the peripheral nervous system (PNS). We demonstrated that following plantar incision, PD-1/PD-L1 is significantly upregulated not in glia, but specifically in CGRP⁺ and IB4⁺ nociceptive neurons within the DRG. It was previously reported that nociceptive sensory are mainly transmitted through IB4^+^ and CGRP^+^ primary afferent neurons^26–29^. CGRP is primarily found in the small- and medium-diameter neurons^30–34^. This neuronal upregulation was associated with attenuated neuroinflammation and pain behaviors, suggesting a previously underappreciated, neuron-intrinsic role for this immunomodulatory pathway.

The localization of PD-1/PD-L1 in sensory neurons points to a direct mechanism for pain control, differing from the indirect, glia-mediated modulation described in CNS pathologies. Our findings suggest that the activation of PD-1 on DRG neurons can autonomously suppress the release of pro-inflammatory cytokines (e.g., TNF-α, IL-1β, IL-6), thereby alleviating peripheral sensitization and acute postoperative pain. This neuron-centric mechanism, complemented by its established role in glial cells, highlights the remarkable cell-type-specific versatility of the PD-1/PD-L1 pathway. Its ability to modulate neuroinflammation and pain across different neural compartments further underscores its potential as a broad-spectrum therapeutic target for diverse pain conditions.

### Limitation

It should be noted that this study focused on the acute phase (24 h) of postoperative pain, when pain behaviors are most pronounced. Future investigations are warranted to determine the sustained role of the PD-1/PD-L1 pathway in the medium-to-long term and its potential in preventing the transition to chronic pain. It is important to acknowledge that this study was conducted exclusively in male rats. This design limits the generalizability of our conclusions regarding the analgesic role of the PD-1/PD-L1 pathway, as significant sex differences in immune modulation and pain processing have been well-documented. Our initial investigation in males was a strategic step to first delineate a clear proof-of-concept mechanism, controlling for the pronounced hormonal fluctuations present in females. Consequently, elucidating whether our findings extend to females, remains an essential and compelling area for future investigation. Finally, our study identifies the functional role of the PD-1/PD-L1 pathway but does not fully unpack the underlying molecular machinery. The key downstream effectors that transduce the PD-1 signal into an anti-inflammatory outcome in sensory neurons represent an important frontier for future work.

## Conclusion

This study demonstrates that activation of the PD-1/PD-L1 signaling pathway alleviates acute postoperative pain in a rat plantar incision model, likely through the suppression of neuroinflammation. Conversely, inhibition of PD-1 exacerbates pain and neuroinflammatory responses. These findings establish the PD-1/PD-L1 axis as a critical endogenous regulator of acute postoperative pain and highlight its potential as a promising therapeutic target for clinical management.

## Supplementary Information

Below is the link to the electronic supplementary material.

Supplementary Material 1

## Data Availability

The data that support the results of this study are available from the corresponding author on reasonable request.

## Declarations

### ARRIVE statement

This study was conducted in accordance with the ARRIVE guidelines.

## References

[1] Glare, P., Aubrey, K. R. & Myles, P. S. Transition from acute to chronic pain after surgery. *Lancet* **393**, 1537–1546 (2019). 30983589 10.1016/S0140-6736(19)30352-6

[2] Richebé, P., Capdevila, X. & Rivat, C. Persistent postsurgical pain: pathophysiology and preventative Pharmacologic considerations. *Anesthesiology* **129**, 590–607 (2018). 29738328 10.1097/ALN.0000000000002238

[3] Vaurio, L. E., Sands, L. P., Wang, Y., Mullen, E. A. & Leung, J. M. Postoperative delirium: the importance of pain and pain management. *Anesth. Analg*. **102**, 1267–1273 (2006). 16551935 10.1213/01.ane.0000199156.59226.af

[4] Ishida, Y., Agata, Y., Shibahara, K. & Honjo, T. Induced expression of PD-1, a novel member of the Immunoglobulin gene superfamily, upon programmed cell death. *EMBO J.* **11**, 3887–3895 (1992). 1396582 10.1002/j.1460-2075.1992.tb05481.x PMC556898

[5] Dong, H., Zhu, G., Tamada, K. & Chen, L. B7-H1, a third member of the B7 family, co-stimulates T-cell proliferation and interleukin-10 secretion. *Nat. Med.* **5**, 1365–1369 (1999). 10581077 10.1038/70932

[6] Polcaro, G. et al. rs822336 binding to C/EBPβ and NFIC modulates induction of PD-L1 expression and predicts anti-PD-1/PD-L1 therapy in advanced NSCLC. *Mol. Cancer*. **23**, 63 (2024). 38528526 10.1186/s12943-024-01976-2 PMC10962156

[7] Luchini, C. et al. ESMO recommendations on microsatellite instability testing for immunotherapy in cancer, and its relationship with PD-1/PD-L1 expression and tumour mutational burden: a systematic review-based approach. *Ann. Oncol.* **30**, 1232–1243 (2019). 31056702 10.1093/annonc/mdz116

[8] Wanderley, C. W. S. et al. PD-1/PD-L1 Inhibition enhances Chemotherapy-Induced neuropathic pain by suppressing neuroimmune antinociceptive signaling. *Cancer Immunol. Res.* **10**, 1299–1308 (2022). 36083496 10.1158/2326-6066.CIR-22-0003

[9] Deng, D. et al. PD-L1/PD-1 pathway: a potential neuroimmune target for pain relief. *Cell. Biosci.* **14**, 51 (2024). 38643205 10.1186/s13578-024-01227-3 PMC11031890

[10] Chen, G. et al. PD-L1 inhibits acute and chronic pain by suppressing nociceptive neuron activity via PD-1. *Nat. Neurosci.* **20**, 917–926 (2017). 28530662 10.1038/nn.4571 PMC5831162

[11] Brennan, T. J., Vandermeulen, E. P. & Gebhart, G. F. Characterization of a rat model of incisional pain. *Pain* **64**, 493–502 (1996). 8783314 10.1016/0304-3959(95)01441-1

[12] Ma, L. et al. STING-IFN-I pathway relieves incision induced acute postoperative pain via inhibiting the neuroinflammation in dorsal root ganglion of rats. *Inflamm. Res.* **72**, 1551–1565 (2023). 37433890 10.1007/s00011-023-01764-6

[13] Deng, D. et al. Electroacupuncture alleviates CFA-Induced inflammatory pain via PD-L1/PD-1-SHP-1 pathway. *Mol. Neurobiol.* **60**, 2922–2936 (2023). 36753045 10.1007/s12035-023-03233-x

[14] Song, X., Zhang, Y., Liu, Y., Chen, G. & Zhao, L. Enhanced analgesic efficacy and reduced side effects of morphine by combination with PD-1 agonist. *ACS Chem. Neurosci.* **16**, 490–499 (2025). 39837575 10.1021/acschemneuro.4c00732

[15] Uçeyler, N. et al. Deficiency of the negative immune regulator B7-H1 enhances inflammation and neuropathic pain after chronic constriction injury of mouse sciatic nerve. *Exp. Neurol.* **222**, 153–160 (2010). 20051242 10.1016/j.expneurol.2009.12.026

[16] Shi, S. et al. PD-L1 and PD-1 expressed in trigeminal ganglia May inhibit pain in an acute migraine model. *Cephalalgia: Int. J. Headache*. **40**, 288–298 (2020). 10.1177/0333102419883374 31640402

[17] Jiang, C. et al. PD-1 regulates GABAergic neurotransmission and GABA-Mediated analgesia and anesthesia. *iScience* **23**, 101570 (2020). 33083737 10.1016/j.isci.2020.101570 PMC7530307

[18] Baxi, S. et al. Immune-related adverse events for anti-PD-1 and anti-PD-L1 drugs: systematic review and meta-analysis. *BMJ (Clinical Res. ed.)*. **360**, k793 (2018). 10.1136/bmj.k793 PMC5851471 29540345

[19] Keir, M. E., Butte, M. J., Freeman, G. J. & Sharpe, A. H. PD-1 and its ligands in tolerance and immunity. *Annu. Rev. Immunol.* **26**, 677–704 (2008). 18173375 10.1146/annurev.immunol.26.021607.090331 PMC10637733

[20] Xiao, M. et al. CD4(+) T-cell epitope-based heterologous prime-boost vaccination potentiates anti-tumor immunity and PD-1/PD-L1 immunotherapy. *Journal for immunotherapy of cancer* 10. (2022). 10.1136/jitc-2021-004022 PMC9114852 35580929

[21] Linnerbauer, M. et al. PD-L1 positive astrocytes attenuate inflammatory functions of PD-1 positive microglia in models of autoimmune neuroinflammation. *Nat. Commun.* **14**, 5555 (2023). 37689786 10.1038/s41467-023-40982-8 PMC10492803

[22] Kummer, M. P. et al. Microglial PD-1 stimulation by astrocytic PD-L1 suppresses neuroinflammation and alzheimer’s disease pathology. *EMBO J.* **40**, e108662 (2021). 34825707 10.15252/embj.2021108662 PMC8672180

[23] Gao, X., Li, W., Syed, F., Yuan, F., Li, P. & Q. Yu PD-L1 signaling in reactive astrocytes counteracts neuroinflammation and ameliorates neuronal damage after traumatic brain injury. *J. Neuroinflamm.* **19**, 43 (2022). 10.1186/s12974-022-02398-x PMC8822654 35135580

[24] Shen, Y. et al. The Immunomodulatory effect of microglia on ECM neuroinflammation via the PD-1/PD-L1 pathway. *CNS Neurosci. Ther.* **28**, 46–63 (2022). 34766463 10.1111/cns.13760 PMC8673706

[25] Cheng, Y. Y., Chen, B. Y., Bian, G. L., Ding, Y. X. & Chen, L. W. Programmed Death-1 deficiency aggravates motor dysfunction in MPTP model of parkinson’s disease by inducing microglial activation and neuroinflammation in mice. *Mol. Neurobiol.* **59**, 2642–2655 (2022). 35142987 10.1007/s12035-022-02758-x

[26] Zhu, T. et al. ALPK1 expressed in IB4-Positive neurons of mice trigeminal ganglions promotes MIA-Induced TMJ pain. *Mol. Neurobiol.* **60**, 6264–6274 (2023). 37442857 10.1007/s12035-023-03462-0

[27] Alvarez, P., Gear, R. W., Green, P. G. & Levine, J. D. IB4-saporin attenuates acute and eliminates chronic muscle pain in the rat. *Exp. Neurol.* **233**, 859–865 (2012). 22206923 10.1016/j.expneurol.2011.12.019 PMC3272112

[28] Pinto, L. G. et al. Non-Peptidergic nociceptive neurons are essential for mechanical inflammatory hypersensitivity in mice. *Mol. Neurobiol.* **56**, 5715–5728 (2019). 30674034 10.1007/s12035-019-1494-5

[29] Tanioku, T. et al. Tmem45b is essential for inflammation- and tissue injury-induced mechanical pain hypersensitivity. *Proc. Natl. Acad. Sci. U.S.A.* **119**, e2121989119 (2022). 36322717 10.1073/pnas.2121989119 PMC9659417

[30] Iyengar, S., Ossipov, M. H. & Johnson, K. W. The role of calcitonin gene-related peptide in peripheral and central pain mechanisms including migraine. *Pain* **158**, 543–559 (2017). 28301400 10.1097/j.pain.0000000000000831 PMC5359791

[31] Porreca, F. et al. Evaluation of outcomes of calcitonin gene-related peptide (CGRP)-targeting therapies for acute and preventive migraine treatment based on patient sex. *Cephalalgia* **44**, 3331024241238153 (2024). 38477313 10.1177/03331024241238153

[32] Thomas, J. L., Schindler, E. A. & Gottschalk, C. Meningeal lymphatic vessel dysfunction driven by CGRP signaling causes migraine-like pain in mice. *J Clin. Invest* **134**. (2024). 10.1172/JCI182556 PMC11290958 39087472

[33] Powell, R. et al. Inhibiting endocytosis in CGRP(+) nociceptors attenuates inflammatory pain-like behavior. *Nat. Commun.* **12**, 5812 (2021). 34608164 10.1038/s41467-021-26100-6 PMC8490418

[34] Xie, S. et al. Monoclonal antibody targeting CGRP relieves Cisplatin-Induced neuropathic pain by attenuating neuroinflammation. *Neurotox. Res.* **42**, 8 (2024). 38194189 10.1007/s12640-023-00685-w

