# Supplementary material for: PD-1/PD-L1 pathway relieves incision induced acute postoperative pain via inhibiting the neuroinflammation in dorsal root ganglion of rats
Source: Sci Rep. 2025 Dec 17;16:2007. doi: 10.1038/s41598-025-31618-6 (PMC12808136; doi:10.1038/s41598-025-31618-6)
Supplement: Supplementary file 1 — Supplementary Material 1 [file 41598_2025_31618_MOESM1_ESM.pdf]

Supplementary material

PD-1/PD-L1 pathway relieves incision induced acute postoperative pain via inhibiting the neuroinflammation in dorsal root ganglion of rats

Honglei Zhang[1, #], Yi Wang[1, #], Shiwei Wen[2], Jing Li[1, *]

1. Department of Anesthesiology, Affiliated Hospital of Zunyi Medical University, 149 Dalian Street, Zunyi, 563000, Guizhou, China.

2. Department of Operating Room, Affiliated Hospital of Zunyi Medical University, 149 Dalian Street, Zunyi, 563000, Guizhou, China.

[#]These authors have contributed equally to this work and share first authorship.

[*]Correspondence: Jing Li, 249972159@qq.com.

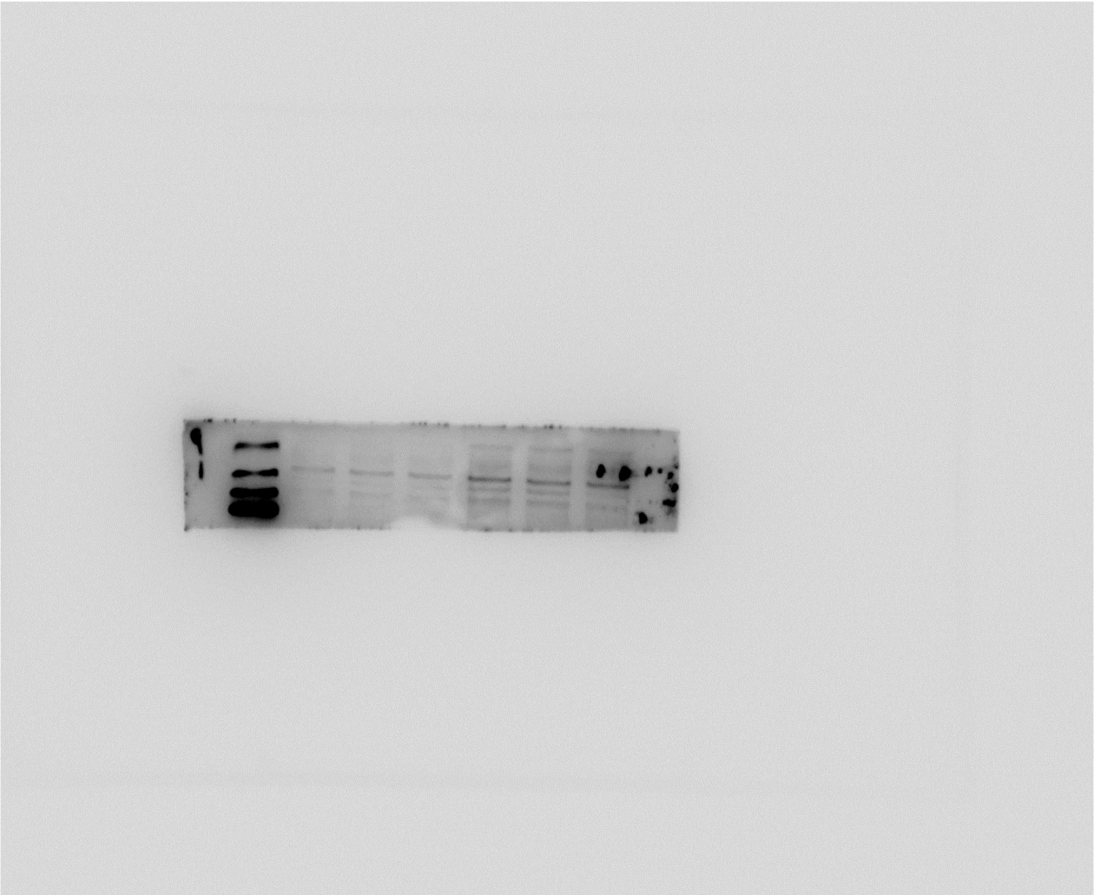

Figure 1D PD-L1

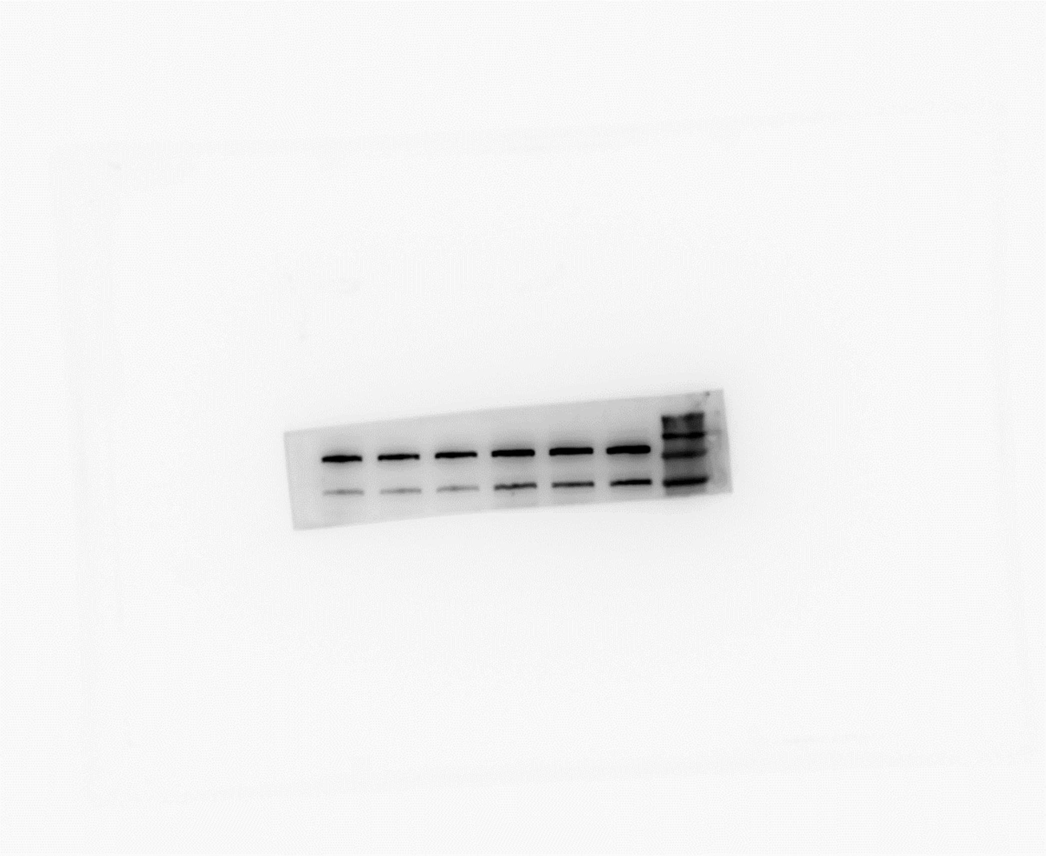

Figure 1D PD-1

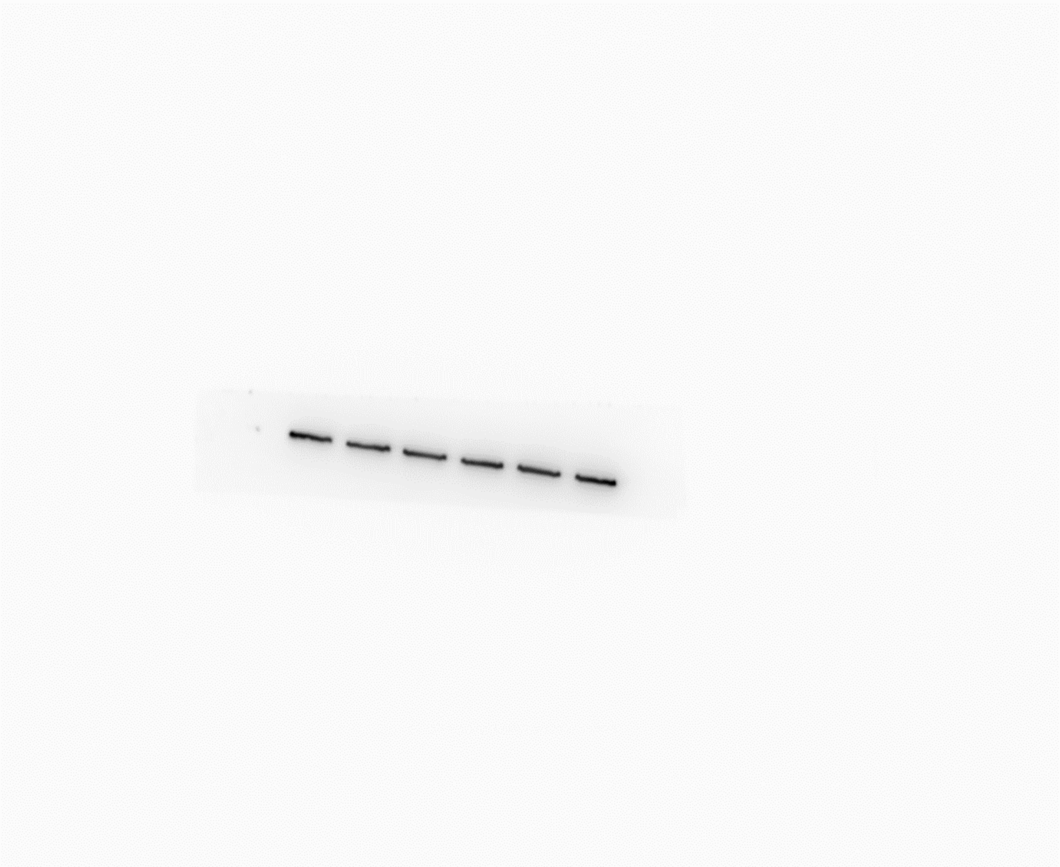

Figure 1D β-actin

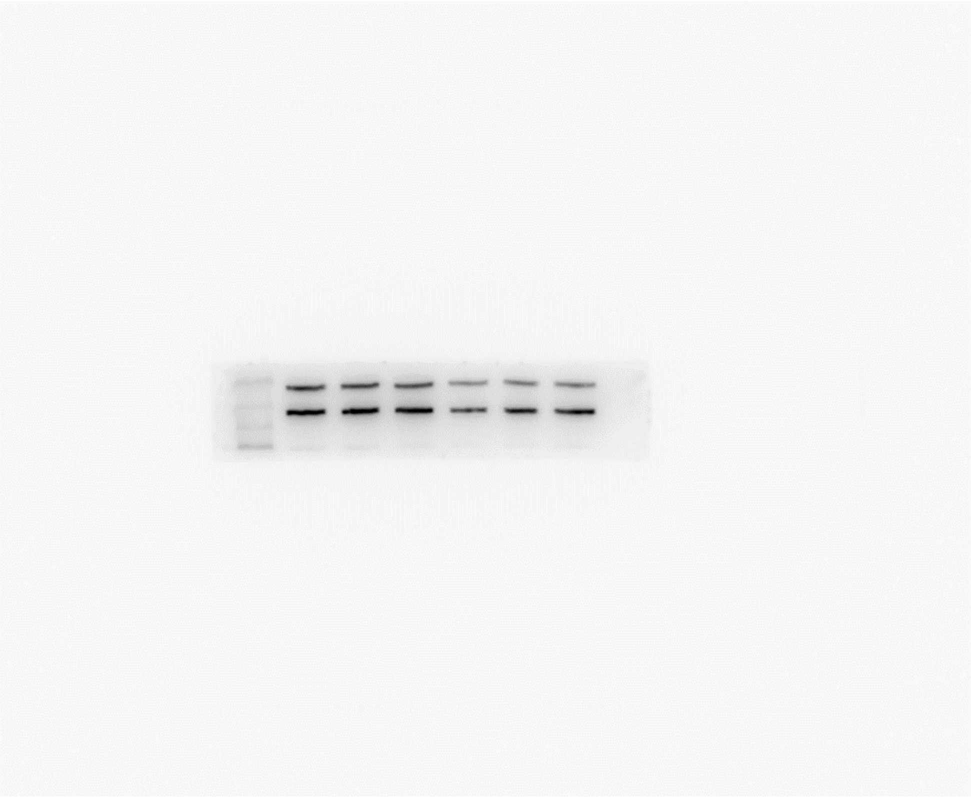

Figure 3D PD-1

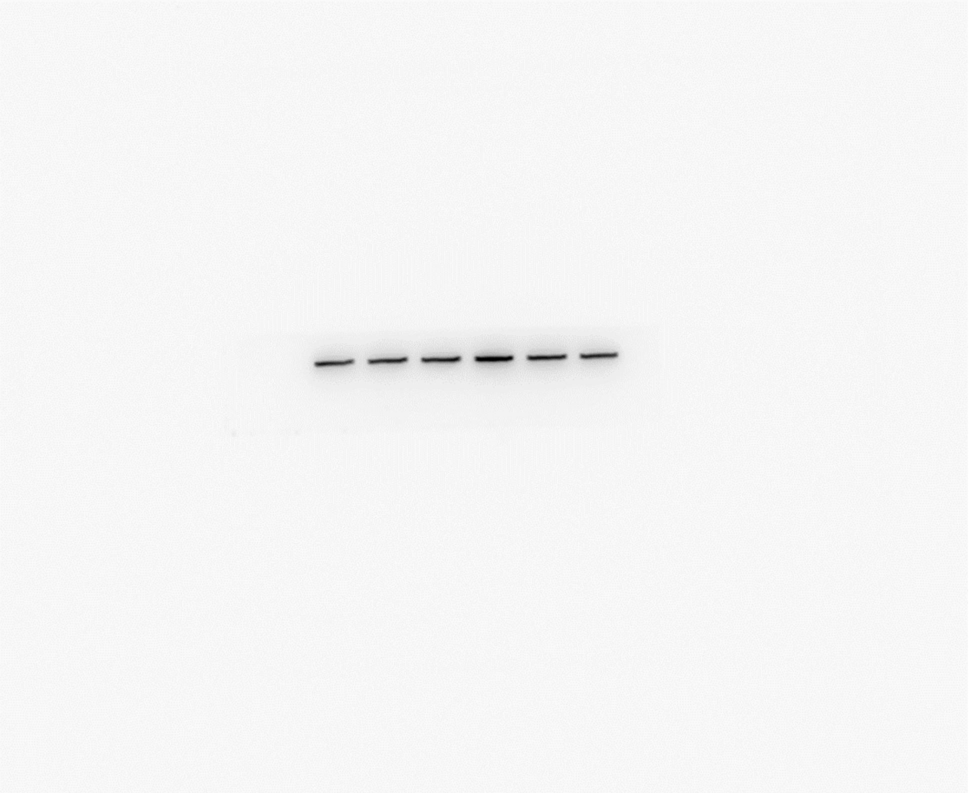

Figure 3D β-actin

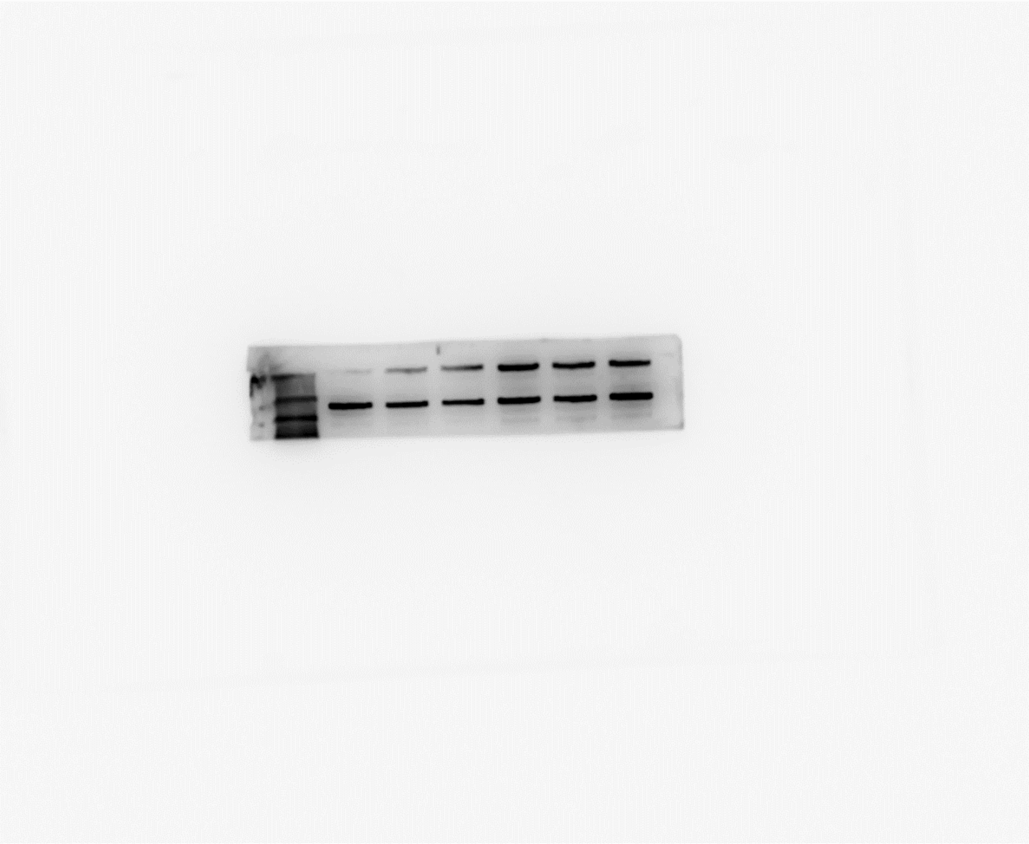

Figure 5D PD-1

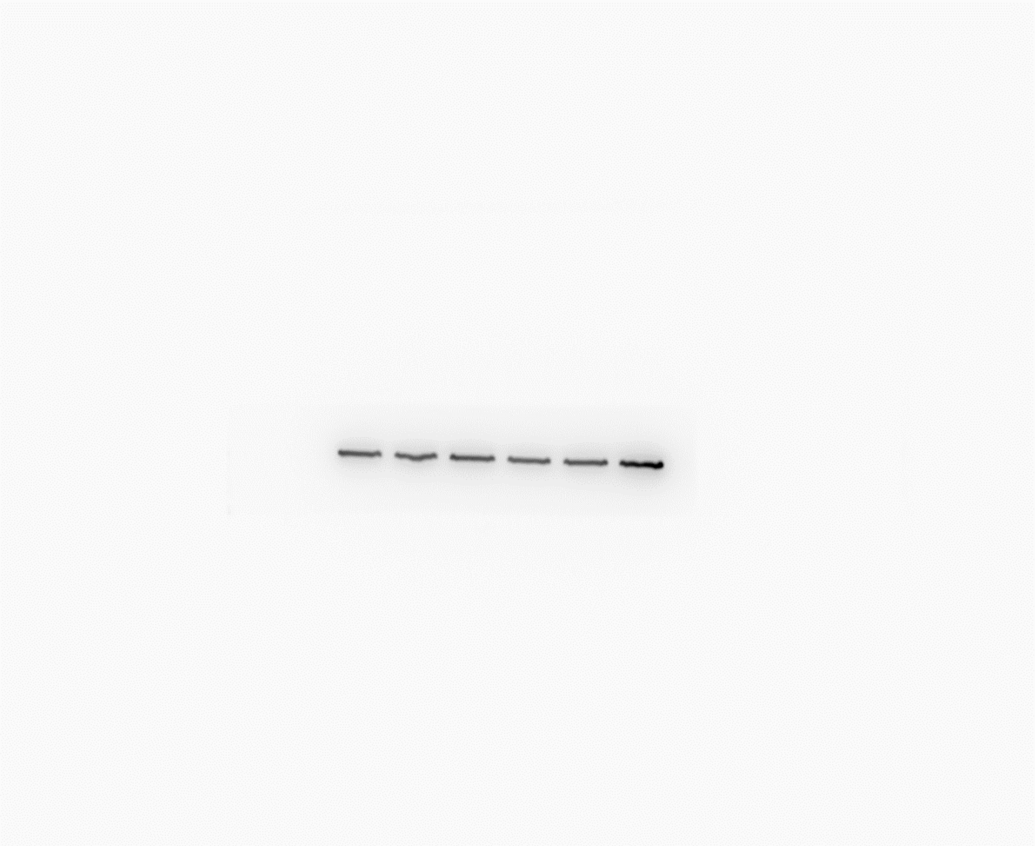

Figure 5D β-actin
